# Application of Variational Method to Stability Analysis of Cantilever Vertical Plates with Bimodular Effect

**DOI:** 10.3390/ma14206129

**Published:** 2021-10-15

**Authors:** Xuan-Yi Xue, Da-Wei Du, Jun-Yi Sun, Xiao-Ting He

**Affiliations:** 1 School of Civil Engineering, Chongqing University, Chongqing 400045, China; xuexuanyi@cqu.edu.cn (X.-Y.X.); 20145190@cqu.edu.cn (D.-W.D.); sunjunyi@cqu.edu.cn (J.-Y.S.); 2 Key Laboratory of New Technology for Construction of Cities in Mountain Area (Chongqing University), Ministry of Education, Chongqing 400045, China

**Keywords:** variational method, stability analysis, cantilever vertical plate, bimodular effect, critical load

## Abstract

In the design of cantilevered balconies of buildings, many stability problems exist concerning vertical plates, in which reaching a critical load plays an important role during the stability analysis of the plate. At the same time, the concrete forming vertical plate, as a typical brittle material, has larger compressive strength but lower tensile strength, which means the tensile and compression properties of concrete are different. However, due to the complexities of such analyses, this difference has not been considered. In this study, the variational method is used to analyze stability problems of cantilever vertical plates with bimodular effect, in which different loading conditions and plate shapes are also taken into account. For the effective implementation of a variational method, the bending strain energy based on bimodular theory is established first, and critical loads of four stability problems are obtained. The results indicate that the bimodular effect, as well as different loading types and plate shapes, have influences on the final critical loads, resulting in varying degrees of buckling. In particular, if the average value of the tensile modulus and compressive modulus remain unchanged, the introduction of the bimodular effect will weaken, to some extent, the bending stiffness of the plate. Among the four stability problems, a rectangular plate with its top and bottom loaded is most likely to buckle; next is a rectangular plate with its top loaded, followed by a triangular plate with its bottom loaded. A rectangular plate with its bottom loaded is least likely to buckle. This work may serve as a theoretical reference for the refined analysis of vertical plates. Plates are made of concrete or similar material whose bimodular effect is relatively obvious and cannot be ignored arbitrarily; otherwise the greater inaccuracies will be encountered in building designs.

## 1. Introduction

**Figure 1:**
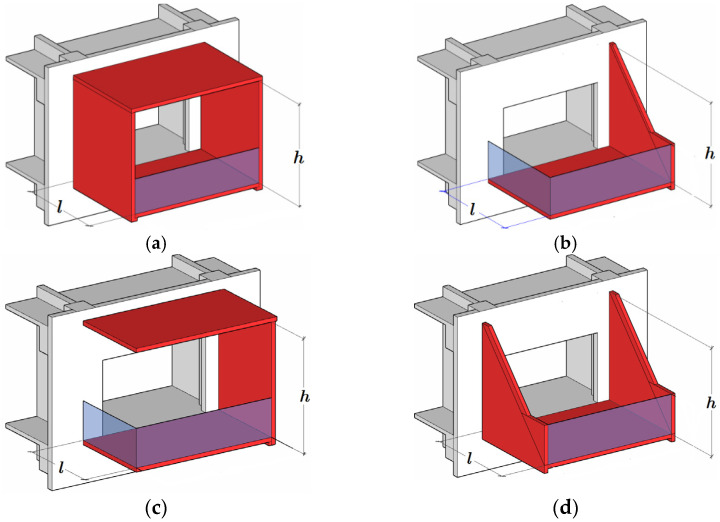
Four structural forms of longer-cantilevered balconies: (**a**) O-shaped, (**b**) L-shaped, (**c**) C-shaped and (**d**) U-shaped.

In structural engineering, the analysis and design of cantilevered balconies are of huge importance, especially for long-cantilevered balconies. Basically, there are four structural forms for long-cantilevered balconies: (a) O-shaped, (b) L-shaped, (c) C-shaped and (d) U-shaped, as shown in Figure 1, in which *h* and *l* are the height and length of the cantilever vertical plate, respectively. In Figure 1, it may be seen that for a cantilever vertical plate, there are two basic shapes: rectangular, as shown in (a) and (c), and triangular, as shown in (b) and (d). In the four structural forms, the rectangular or triangular plate serves as an important structural element in resisting external loads from the top and bottom of the balcony; therefore, it is a key load-bearing component in cantilevered balconies. Among static, dynamic and stability analyses for cantilever vertical plates, the stability problem is particularly prominent because the vertical plate must reach a certain height if it is to meet the design and functional requirements of the building’s architecture; compressive stress in the plate becomes inevitable, inducing elastic buckling due to the relatively low plate thickness. On the other hand, most parts constituting the balcony plate are made of concrete, which has greater compressive strength but lower tensile strength. This disparity between tensile and compressive strength also seems to mean that the tensile and compressive properties of concrete are different. This creates an issue of concern, i.e., what are the effects of different types and degrees of tension and compression? Therefore, this paper provides a stability analysis of cantilever vertical plates from a long-cantilevered balcony in which the applied material has different properties in terms of tension and compression. We will first discuss the elastic buckling problems of plates and the corresponding solutions, before addressing the bimodular problem, as discussed in existing studies.

The elastic buckling problem associated with plates has been extensively investigated over the last century. All kinds of plate shapes, different boundary conditions and applied in-plane force distributions have been considered, and buckling critical loads are documented in some stability handbooks [1], standard texts on plates [2,3,4] and in a large number of technical papers (for example, [5,6]). Under different boundary constraints, Wang et al. [7] investigated the elastic buckling problem of vertical plates under body forces (self-weight) in which the extensive buckling body force parameters presented were shown to be useful to engineers for designing structures or machines with vertical plate components. Wang [8] studied the buckling of a standing plate subjected to self-weight and top load, in which an initial value method which was accurate, and more efficient than previous methods, was used. His results indicated that the effects of self-weight and top load both contribute to buckling, although their contributions are not linear. Notably, for narrow plates, if top load dominates, it does not matter whether an end is clamped or simply supported, while if self-weight dominates, the buckling of a narrow plate depends only on the bottom condition. The buckling problems of rectangular plates with various boundary conditions under nonuniform, in-plane loads were investigated in [9,10,11]. It was found that the critical buckling load is associated with loading form and constraint conditions. Jędrysiak and Michalak [12] dealt with the stability problem of thin composite plates with a smooth and slow gradation of macroscopic properties. In their study, governing equations of the tolerance models for the stability of thin plates with functionally graded structures were derived. Chróścielewski [13] investigated the torsional buckling phenomenon of thin-walled I-beam columns. In their study, the effect of initial deflection on the torsional buckling load of a thin-walled I-beam column was discussed, and the localization of local buckling modes was studied. Magnucka-Blandzi [14] studied the bending and buckling of a circular plate with symmetrically varying mechanical properties. Magnucki and Magnucka-Blandzi [15] generalized the model of sandwich structure, in which analytical studies of bending and buckling problems of rectangular plates were presented. Through an experiment and numerical simulation, Zhang et al. [16] investigated the compressive buckling behavior of a double-sided, laser-welded Al–Li alloy aircraft fuselage panel. Kolakowski and Jankowski [17,18] investigated the effect of the membrane components of transverse forces on the magnitudes of total transverse forces in the nonlinear stability of plate structures, and noted some inconsistencies in nonlinear buckling in first-order and higher-order shear deformation theory. Cheng [19,20] applied a variational method to analyze the lateral instability problem of cantilever rectangular plates, obtaining the critical loads of plates under the action of a concentrated force, uniformly-distributed loads, triangularly-distributed loads and a concentrated couple. His results indicated that the values of the critical loads are associated with the bending stiffness of the plate and the aspect ratio of rectangular plane, as well as with the choice of the corresponding deflection function. In this study, we focus on determining the critical buckling load. Therefore, the variational method is applied.

Among the various methods of solving the stability problem of plates, there are basically three which apply analytical techniques. The first is the triangle series expansion method, which first requires the buckling differential equation of the plate to be expressed in terms of the deflection and internal forces, and then yields the associated internal forces. The use of this method is limited as, in some cases, it is difficult to establish the buckling differential equation. The second is the differential method, which also makes use of the buckling differential equation. The third is the variational method based on the law of energy, which only requires knowledge of the bending strain energy of the plate and the work done by external forces, thus avoiding the need to include the buckling differential equation. Although the variational method can be used to determine the critical load only, and the displacement and internal forces under the critical load remain unknown, it is still believed that the method may serve as an effective way to determine critical loads, which is particularly important in analyses of plate stability, while the displacement and internal forces are generally obtained as secondary elements, especially in the design of plate.

On the other hand, the structural material also plays an important role in the analysis and design of the structural elements. Generally, the structural material forming the vertical plates in cantilevered balconies is concrete, which is regarded as continuous, elastic, homogeneous and isotropic. However, many studies have indicated that most materials, including ceramics, plastics, concrete, graphite, powder metallurgy materials, polymeric materials and some composites exhibit different tensile and compressive strains under the same tension or compression stress [21,22]. Thus, certain materials exhibit different elastic moduli for tension and compression; these materials are referred to as “bimodular” materials [23]. The concrete used in the present analysis, without exception, is a bimodular material. As a brittle material, the concrete forming vertical plates possesses larger compressive strength but lower tensile strength, which means that its tensile and compression properties are different. Up to now, few reports have been published on concrete with a bimodular effect, even though consideration of the bimodular effect of structural concrete is crucial.

Overall, two bimodular material models are widely used in theoretical analyses within the field of engineering. One is the criterion of positive–negative signs in the longitudinal strain of fibers, as proposed by Bert [24]. This model is mainly applicable to orthotropic materials, and is therefore widely used for research on laminated composites [25,26,27,28]. The other model is the criterion of positive–negative signs of principal stress put forward by Ambartsumyan [29], which is mainly applicable to isotropic materials. In structural engineering, the stress state along certain principal directions is a key issue in stress analyses of components like beams and plates, since it is this factor that determines whether the point is under tension or compression. Due to the fact that this bimodular theory defines the constitutive model based on principal directions, and the principal stress is generally obtained as the final result but not as a known condition before solving, this inevitably gives rise to difficulties in terms of describing the stress state of a given point. This model also lacks the ability to describe the experimental results of elastic coefficients in complex states of stress. Analytical solutions are available in a few cases, although they only concern the static, dynamic and thermal problems of beams and plates [30,31,32,33,34]. In some complex problems, it is necessary to resort to a finite element method (FEM) based on an iterative technique [35,36,37,38].

To date, numerous studies have been published concerning the stability problem for plates under different loads. However, for vertical plates, there is significantly less reported research. From the literature we collected, reports concerning vertical plates mainly included the elastic buckling problem of vertical plates under body forces (self-weight) [7], the buckling of a standing plate subjected to self-weight and top load [8], the lateral instability problem of cantilever rectangular plates under the action of concentrated force, uniformly-distributed loads, triangularly-distributed loads and concentrated couple [19], and the buckling of cantilever rectangular plates under symmetrical edge loading [20]. Among these works, there are obviously two aspects that could be improved upon. One is the fact that none of these studies took into account the bimodular effect of the material, which seems to be problematic. If the material possesses a significant bimodular effect, the error due to the absence of this parameter will be significant. Another may come from the plate shape. For example, triangular plates are seldom discussed (N.B. the originality of the present study is not constituted by our inclusion of triangular plates alone). Broadly speaking, investigations of the stability problems of cantilever vertical plates with different loading types and plate shapes, also considering the bimodular effect of the materials, will be helpful for future analyses of the buckling problem of plates from the perspective of design optimization.

In this study, the variational method is used to solve the stability problem of cantilever vertical plates with bimodular effect, in which different loading types and plate shapes which are widely applied in the construction of real balconies are considered. This paper is arranged as follows. In Section 2, four stability problems of plates with bimodular effect are presented. For the purpose of the effective implementation of the variational method based on bimodular theory, in Section 3, the strain potential energy of a bimodular plate is derived and the work done by external loads is presented. In Section 4, the variational method is applied to determine the critical loads in the four stability problems. The four critical loads without bimodular effect are discussed and the bimodular effect on critical loads is analyzed in Section 5. Some important conclusions are drawn in Section 6.

## 2. Stability Problem of Bimodular Cantilever Vertical Plates

**Figure 2:**
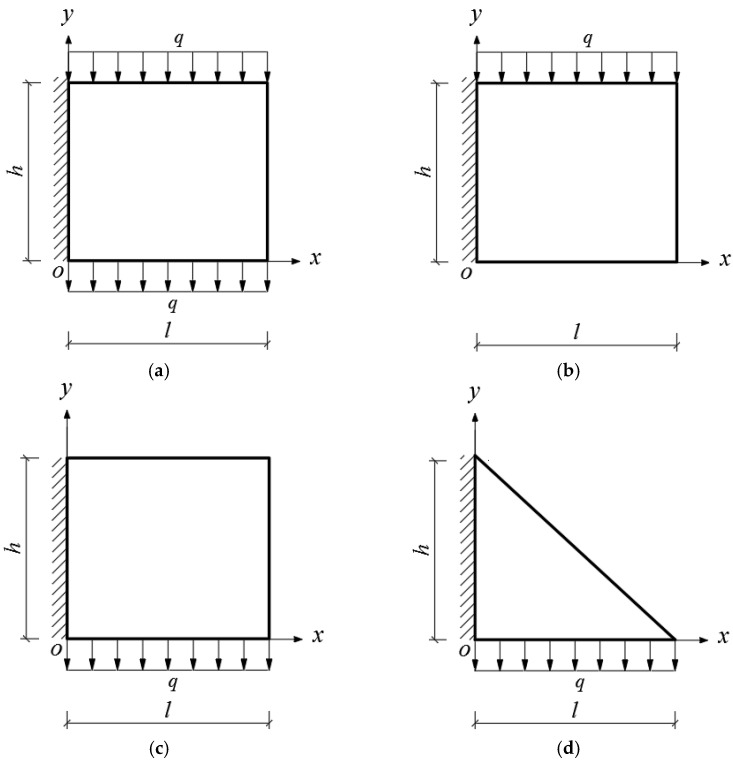
Four stability problems with bimodular effect: (**a**) Rectangular cantilever vertical plate with top and bottom loaded, (**b**) Rectangular cantilever vertical plate with top loaded, (**c**) Rectangular cantilever vertical plate with bottom loaded and (**d**) Triangular cantilever vertical plate with bottom loaded.

Given the structural forms of the balconies presented in Figure 1, two rectangular and two triangular types of cantilever vertical plates are analyzed, as shown in Figure 2. The cantilever length of the plate and the height of the plate are denoted by *l* and *h*, respectively. In Figure 2, the *xoy* plane coordinate system is established, and *o* is the origin of the coordinate system. According to the force transmission characteristic of the components, the loads are transferred from top to bottom in turn, so that all the loads act downward. Among the stability problems shown in Figure 2, Case (a) is typical, since the vertical plate is subjected to loads from the top and bottom simultaneously. Cases (b) and (c) are supplementary problems, intended to serve as comparisons with Case (a). Comparing Figure 1 and Figure 2, it is easy to see that Cases (a), (b) and (c) in Figure 2 correspond to the O-shaped and C-shaped balconies in Figure 1, while Case (d) corresponds to the L-shaped and U-shaped balconies in Figure 1, in which the cantilever vertical plate is triangular, with its bottom loaded only. For simplification and ease of comparison, we regard all loads *q* as being uniformly distributed, and for Cases (a), the magnitudes of the two loads are the same when the vertical plate is under the combined action of the top and bottom loads.

Additionally, the vertical plate is made of concrete which, in the analysis, is treated as continuous, elastic, isotropic and homogenous, but with the bimodular characteristic indicated above. Generally, in reality, the concrete is reinforced by steel bars in order to make it more resistant to tensile force. In the theoretical analysis presented here, however, we only consider plain concrete without reinforcements. Therefore, our results may serve as a theoretical reference for subsequent reinforcements. Alternatively, from the point of view of structural design, the present theoretical analysis is based on plain concrete and any reinforcements are considered as a safety reserve.

## 3. Variational Method Based on Bimodular Materials Theory

The total potential energy of the system is

(1) ∏=U−W,

where *U* and *W* are the strain potential energy and the potential of external loads, respectively. From the point of view of energy, the critical load can be obtained on condition that the work done by the variation of external loads, δ*W*, is equal to the variation of strain potential energy, δ*U*, when the plate deforms from the in-plane state to the adjacent bending state. According to Rayleigh-Ritz method, when the system reaches the limit of stable equilibrium, the total potential energy is the minimum, that is

(2) δ∏=0,

which may be used to determine the critical load. It should be noted that in the application of this variational method, *U* and *W* must be expressed in terms of the *z*-axis displacement, *w*, and that *w* should satisfy all boundary conditions of displacement. As for the stress boundary conditions, *w* need not be satisfied, but if a part or all of it can be satisfied, the solution accuracy is greatly improved. In addition, we also note that the calculation of *U* is based on the theory of elasticity from uniform modulus. If the bimodular theory of elasticity is introduced here, some important improvements may be incorporated. In determining *W*, however, the bimodular effect has no any influence.

### 3.1. Strain Potential Energy of Bimodular Plates

First, let us derive the expression of the increase of strain potential energy *U*, which may originate from two different aspects. The first is from the bending deformation in the vertical plane; however, this is negligible, since the bending stiffness in the vertical plane is very large. The second is the torsion and transverse bending under the critical load, thus making the plate deviate from its original vertical plane. The potential energy generated by this deformation represents the vast majority of the total potential energy, so we only consider the deformation of torsion and bending when calculating the increase of potential energy. This practice is similar to the buckling problem of the straight bar in compression, in which only the bending strain energy is considered, while the strain energy from axial compression deformation is ignored.

**Figure 3:**
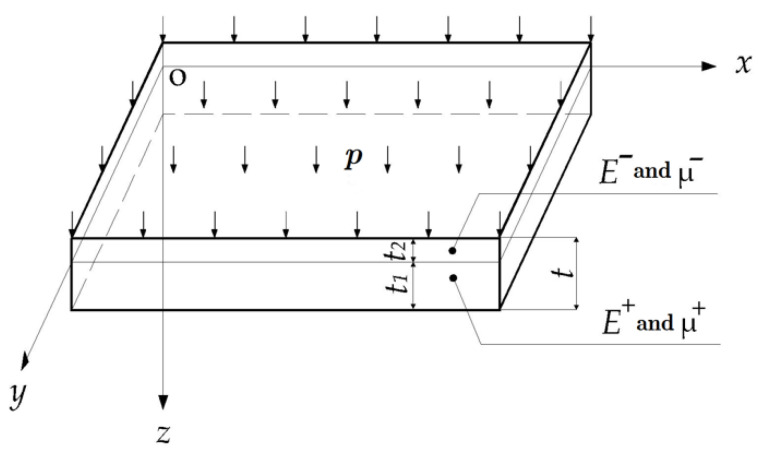
Bending analysis of a bimodular rectangular thin plate.

Figure 3 shows a rectangular thin plate with different moduli under tension and compression which is being subjected to the vertical downward uniformly-distributed loads, *p*, and which is thus bending downward and forming the tensile and compressive areas which are bounded by an unknown neutral layer. In Figure 3, the *xoy* plane is established on the unknown neutral layer and the *z* axis is vertical. The thickness of the plate is *t*, while *t*_1_ and *t*_2_ stand for the tensile and compressive thickness, respectively, both of which will be determined later. *E^+^* and *μ^+^* are the tensile Young’s modulus of elasticity and Poisson ratio, respectively; *E*^−^ and *μ*^−^ are the compressive modulus and Poisson ratio, as shown in Figure 3. The constraints of the four sides of the plate are variable; for example, four sides may be fixed or simply-supported, two opposite sides may be fixed or simply-supported, or other, mixed constraint modes may be applied, so long as they cause downward bending under uniformly-distributed load, *p*.

Based on the Kirchhoff-Love hypothesis, the small-deflection bending problem of a thin plate may be analyzed by two-dimensional thin plate bending theory, in which the physical equation will adopt the equation in the state of plane stress, neglecting the effects of the strain components *ε_z_*, *γ_yz_* and *γ_zx_*. Meanwhile, the introduction of the bimodular effect of the material has no influence on the geometrical relation based on deformation characteristics, and therefore, the geometrical equation can still be written as

(3) $$\left\{ \begin{array}{l} \varepsilon_{x}=-\frac{\partial^{2}w}{\partial x^{2}}z\\ \varepsilon_{y}=-\frac{\partial^{2}w}{\partial y^{2}}z\\ \gamma_{xy}=-2\frac{\partial^{2}w}{\partial x\partial y}z \end{array}\right.,$$

where *w* is the deflection along the *z* axis, and *ε_x_*, *ε_y_* and *γ_xy_* are the strain components in two-dimensional thin plate bending theory. Note that due to subarea under tension and compression, the stress components in the tensile and compressive areas are different, which may be expressed as $\sigma_{x}^{+},\sigma_{y}^{+},\tau_{xy}^{+}$ for the tensile area and $\sigma_{x}^{-},\sigma_{y}^{-},\tau_{xy}^{-}$ for the compressive area. Thus the physical equation in the state of plane stress in the tensile area (0 *≤ z ≤ t*_1_,) may be given as

(4) $$\left\{ \begin{array}{l} \sigma_{x}^{+}=\frac{E^{+}}{1-{\left(\mu^{+}\right)}^{2}}(\varepsilon_{x}+\mu^{+}\varepsilon_{y})\\ \sigma_{y}^{+}=\frac{E^{+}}{1-{\left(\mu^{+}\right)}^{2}}(\varepsilon_{y}+\mu^{+}\varepsilon_{x})\\ \tau_{xy}^{+}=\frac{E^{+}}{2(1+\mu^{+})}\gamma_{xy} \end{array}\right.,$$

and in the compressive area (−*t*_2_ *≤ z ≤* 0) as

(5) $$\left\{ \begin{array}{l} \sigma_{x}^{-}=\frac{E^{-}}{1-{\left(\mu^{-}\right)}^{2}}(\varepsilon_{x}+\mu^{-}\varepsilon_{y})\\ \sigma_{y}^{-}=\frac{E^{-}}{1-{\left(\mu^{-}\right)}^{2}}(\varepsilon_{y}+\mu^{-}\varepsilon_{x})\\ \tau_{xy}^{-}=\frac{E^{-}}{2(1+\mu^{-})}\gamma_{xy} \end{array}\right..$$

Substituting Equation (3) into Equations (4) and (5), for 0 *≤ z ≤ t*_1_, we have

(6) $$\left\{ \begin{array}{l} \sigma_{x}^{+}=-\frac{E^{+}z}{1-{\left(\mu^{+}\right)}^{2}}\left(\frac{\partial^{2}w}{\partial x^{2}}+\mu^{+}\frac{\partial^{2}w}{\partial y^{2}}\right)\\ \sigma_{y}^{+}=-\frac{E^{+}z}{1-{\left(\mu^{+}\right)}^{2}}\left(\frac{\partial^{2}w}{\partial y^{2}}+\mu^{+}\frac{\partial^{2}w}{\partial x^{2}}\right)\\ \tau_{xy}^{+}=-\frac{E^{+}z}{1+\mu^{+}}\frac{\partial^{2}w}{\partial x\partial y} \end{array}\right.,$$

and for −*t*_2_ *≤ z ≤* 0,

(7) $$\left\{ \begin{array}{l} \sigma_{x}^{-}=-\frac{E^{-}z}{1-{\left(\mu^{-}\right)}^{2}}\left(\frac{\partial^{2}w}{\partial x^{2}}+\mu^{-}\frac{\partial^{2}w}{\partial y^{2}}\right)\\ \sigma_{y}^{-}=-\frac{E^{-}z}{1-{\left(\mu^{-}\right)}^{2}}\left(\frac{\partial^{2}w}{\partial y^{2}}+\mu^{-}\frac{\partial^{2}w}{\partial x^{2}}\right)\\ \tau_{xy}^{-}=-\frac{E^{-}z}{1+\mu^{-}}\frac{\partial^{2}w}{\partial x\partial y} \end{array}\right..$$

The bending moment per unit length on the section normal to *x* and *y* axes is *M_x_* and *M_y_*, respectively. These values may be computed by integrating the segments of the tensile and compressive thicknesses, as follows:

(8) $$\begin{array}{ll} M_{x} & =\int_{0}^{t_{1}}\sigma_{x}^{+}zdz+\int_{-t_{2}}^{0}\sigma_{x}^{-}zdz\\ & =-\frac{E^{+}t_{1}^{3}}{3[1-{\left(\mu^{+}\right)}^{2}]}\left(\frac{\partial^{2}w}{\partial x^{2}}+\mu^{+}\frac{\partial^{2}w}{\partial y^{2}}\right)-\frac{E^{-}t_{2}^{3}}{3[1-{\left(\mu^{-}\right)}^{2}]}\left(\frac{\partial^{2}w}{\partial x^{2}}+\mu^{-}\frac{\partial^{2}w}{\partial y^{2}}\right) \end{array}$$

and

(9) $$\begin{array}{ll} M_{y} & =\int_{0}^{t_{1}}\sigma_{y}^{+}zdz+\int_{-t_{2}}^{0}\sigma_{y}^{-}zdz\\ & =-\frac{E^{+}t_{1}^{3}}{3[1-{\left(\mu^{+}\right)}^{2}]}\left(\frac{\partial^{2}w}{\partial y^{2}}+\mu^{+}\frac{\partial^{2}w}{\partial x^{2}}\right)-\frac{E^{-}t_{2}^{3}}{3[1-{\left(\mu^{-}\right)}^{2}]}\left(\frac{\partial^{2}w}{\partial y^{2}}+\mu^{-}\frac{\partial^{2}w}{\partial x^{2}}\right) \end{array}$$

Additionally, the torsion moment per unit length on the section normal to *x* axis, *M_xy_*, is

(10) $$M_{xy}=\int_{0}^{t_{1}}\tau_{xy}^{+}zdz+\int_{-t_{2}}^{0}\tau_{xy}^{-}zdz=-\frac{1}{3}\left(\frac{E^{+}t_{1}^{3}}{1+\mu^{+}}+\frac{E^{-}t_{2}^{3}}{1+\mu^{-}}\right)\frac{\partial^{2}w}{\partial x\partial y}.$$

In the small-deflection bending problem of thin plates under the action of the uniformly-distributed load, *p*, the equation of equilibrium expressed in terms of the bending moments, *M_x_* and *M_y_*, and the torsion moment, *M_xy_*, gives

(11) $$\frac{\partial^{2}M_{x}}{\partial x^{2}}+2\frac{\partial^{2}M_{xy}}{\partial x\partial y}+\frac{\partial^{2}M_{y}}{\partial y^{2}}+p=0.$$

Substituting Equations (8) to (10) into Equation (11), we have

(12) $$\left\{\frac{E^{+}t_{1}^{3}}{3[1-{\left(\mu^{+}\right)}^{2}]}+\frac{E^{-}t_{2}^{3}}{3[1-{\left(\mu^{-}\right)}^{2}]}\right\}\nabla^{4}w=p,$$

where ▽^4^ is the biharmonic operator. If we let *D* be the bending stiffness of a bimodular plate, then

(13) $$D=\frac{E^{+}t_{1}^{3}}{3[1-{\left(\mu^{+}\right)}^{2}]}+\frac{E^{-}t_{2}^{3}}{3[1-{\left(\mu^{-}\right)}^{2}]}.$$

A familiar form may be obtained, such as *D*▽^4^ *w* = *p*.

The strain potential energy *U* in a two-dimensional thin plate with bimodular effect may be written, by neglecting the effects of strain components *ε_z_*, *γ_yz_* and *γ_zx_*, as

(14) $$U=\frac{1}{2}\int \int \int (\sigma_{x}^{+}\varepsilon_{x}+\sigma_{y}^{+}\varepsilon_{y}+\tau_{xy}^{+}\gamma_{xy})dxdydz+\frac{1}{2}\int \int \int (\sigma_{x}^{-}\varepsilon_{x}+\sigma_{y}^{-}\varepsilon_{y}+\tau_{xy}^{-}\gamma_{xy})dxdydz.$$

Substituting Equation (3) as well as Equations (6) and (7) into Equation (14), and also integrating *z*, in which 0 *≤ z ≤ t*_1_ for the first tensile term and −*t*_2_ *≤ z ≤* 0 for the second compressive term, we obtain

(15) $$\begin{array}{ll} U & =\frac{E^{+}}{2[1-{\left(\mu^{+}\right)}^{2}]}\int_{0}^{t_{1}}z^{2}dz\int \int \left[{\left(\frac{\partial^{2}w}{\partial x^{2}}\right)}^{2}+{\left(\frac{\partial^{2}w}{\partial y^{2}}\right)}^{2}+2\mu^{+}\frac{\partial^{2}w}{\partial x^{2}}\frac{\partial^{2}w}{\partial y^{2}}+2(1-\mu^{+}){\left(\frac{\partial^{2}w}{\partial x\partial y}\right)}^{2}\right]dxdy\\ & +\frac{E^{-}}{2[1-{\left(\mu^{-}\right)}^{2}]}\int_{-t_{2}}^{0}z^{2}dz\int \int \left[{\left(\frac{\partial^{2}w}{\partial x^{2}}\right)}^{2}+{\left(\frac{\partial^{2}w}{\partial y^{2}}\right)}^{2}+2\mu^{-}\frac{\partial^{2}w}{\partial x^{2}}\frac{\partial^{2}w}{\partial y^{2}}+2(1-\mu^{-}){\left(\frac{\partial^{2}w}{\partial x\partial y}\right)}^{2}\right]dxdy \end{array}.$$

Lastly, we have

(16) $$\begin{array}{ll} U & =\frac{D^{+}}{2}\int \int \left[{\left(\frac{\partial^{2}w}{\partial x^{2}}\right)}^{2}+{\left(\frac{\partial^{2}w}{\partial y^{2}}\right)}^{2}+2\mu^{+}\frac{\partial^{2}w}{\partial x^{2}}\frac{\partial^{2}w}{\partial y^{2}}+2(1-\mu^{+}){\left(\frac{\partial^{2}w}{\partial x\partial y}\right)}^{2}\right]dxdy\\ & +\frac{D^{-}}{2}\int \int \left[{\left(\frac{\partial^{2}w}{\partial x^{2}}\right)}^{2}+{\left(\frac{\partial^{2}w}{\partial y^{2}}\right)}^{2}+2\mu^{-}\frac{\partial^{2}w}{\partial x^{2}}\frac{\partial^{2}w}{\partial y^{2}}+2(1-\mu^{-}){\left(\frac{\partial^{2}w}{\partial x\partial y}\right)}^{2}\right]dxdy \end{array},$$

where

(17) $$D^{+}=\frac{E^{+}t_{1}^{3}}{3[1-{\left(\mu^{+}\right)}^{2}]},\;D^{-}=\frac{E^{-}t_{2}^{3}}{3[1-{\left(\mu^{-}\right)}^{2}]}.$$

In fact, the total bending stiffness of the bimodular plate is *D = D^+^* *+D*^−^. Upon returning to the uniform modulus problem, we have *E^+^ = E*^−^ *= E*, *μ^+^* *=μ*^−^ *=μ*, and *t*_1_ = *t*_2_ = *t*/2; thus, *D^+^ + D*^−^ *=* *Et*^3^/[24(1 *− μ*^2^)] + *Et*^3^/[24(1 *− μ*^2^)] = *Et*^3^/[12(1 *− μ*^2^)], which satisfies the regression of the solution.

Note that up to now, the tensile section thickness and the compressive one have not been determined. To address this, we use the condition that the normal forces per unit length on the section normal to *x* and *y* axes, *N_x_* and *N_y_*, are zero in order to determine the location of the unknown neutral layer, that is,

(18) $$N_{x}=\int_{0}^{t_{1}}\sigma_{x}^{+}dz+\int_{-t_{2}}^{0}\sigma_{x}^{-}dz=0$$

and

(19) $$N_{y}=\int_{0}^{t_{1}}\sigma_{y}^{+}dz+\int_{-t_{2}}^{0}\sigma_{y}^{-}dz=0.$$

Substituting $\sigma_{x}^{+},\sigma_{y}^{+}$ and $\sigma_{x}^{-},\sigma_{y}^{-}$ from Equations (6) and (7) into Equations (18) and (19), respectively, we have

(20) $$-\frac{E^{+}t_{1}^{2}}{2[1-{\left(\mu^{+}\right)}^{2}]}\left(\frac{\partial^{2}w}{\partial x^{2}}+\mu^{+}\frac{\partial^{2}w}{\partial y^{2}}\right)+\frac{E^{-}t_{2}^{2}}{2[1-{\left(\mu^{-}\right)}^{2}]}\left(\frac{\partial^{2}w}{\partial x^{2}}+\mu^{-}\frac{\partial^{2}w}{\partial y^{2}}\right)=0$$

and

(21) $$-\frac{E^{+}t_{1}^{2}}{2[1-{\left(\mu^{+}\right)}^{2}]}\left(\frac{\partial^{2}w}{\partial y^{2}}+\mu^{+}\frac{\partial^{2}w}{\partial x^{2}}\right)+\frac{E^{-}t_{2}^{2}}{2[1-{\left(\mu^{-}\right)}^{2}]}\left(\frac{\partial^{2}w}{\partial y^{2}}+\mu^{-}\frac{\partial^{2}w}{\partial x^{2}}\right)=0.\;$$

By adding the left end and right end of Equations (20) and (21), we obtain the following relation after simplification

(22) $$\frac{E^{+}t_{1}^{2}}{1-\mu^{+}}=\frac{E^{-}t_{2}^{2}}{1-\mu^{-}}.$$

Combining the relation *t*_1_ + *t*_2_ *= t*, we have

(23) $$\frac{t_{1}}{t}=\frac{\sqrt{E^{-}(1-\mu^{+})}}{\sqrt{E^{+}(1-\mu^{-})}+\sqrt{E^{-}(1-\mu^{+})}}{,}_{}^{}\frac{t_{2}}{t}=\frac{\sqrt{E^{+}(1-\mu^{-})}}{\sqrt{E^{+}(1-\mu^{-})}+\sqrt{E^{-}(1-\mu^{+})}},$$

which determines the location of the unknown neutral layer. Obviously, when *E^+^ = E*^−^ *= E*, *μ^+^* *= μ*^−^ *=* *μ*, the relation *t*_1_ = *t*_2_ = *t*/2, may be easily obtained. Finally, we obtain the expressions of the strain potential energy and the neutral layer of a bimodular plate.

### 3.2. Work Done by External Loads

**Figure 4:**
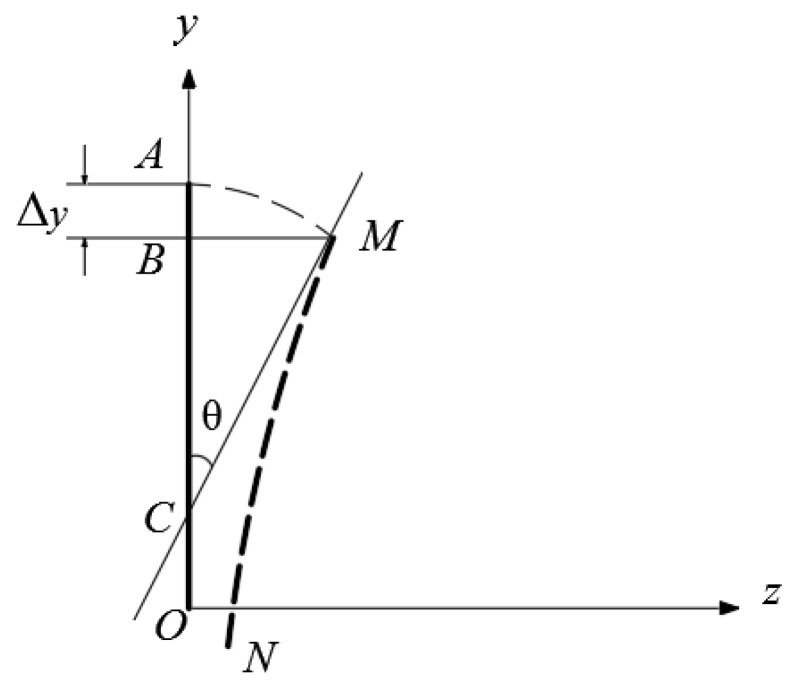
Scheme of differential body and computation of Δ*_y_(x)*.

Next, let us derive the work done by external loads, *W*. From Figure 2, it is easy to see that the external loads are uniformly-distributed, *q*. Supposing the uniformly-distributed loads are acting on the top of the plate, that is *y = h*, the work done by external loads, *W*, may be given as

(24) $$W=\int_{0}^{l}q\Delta_{y}(x)dx,$$

where Δ*_y_*(*x*) is the displacement of the action point of load *q* when the plate undergoes lateral bending. To calculate Δ*_y_*(*x*), we may refer to Figure 4.

In Figure 4, any cross section of the plate represents a derivation along the *z* axis, that is, the plate thickness direction from *AO* to *MN*, where the tangent at point *M* intersects the *y* axis at point *C*, and *θ* is the angle between lines *AC* and *MC*. Note that *θ* is a small quantity, and thus, we have the relations *AC = MC*, *θ =* (*əw/əy*)*_M_* and *MB = w_M_*, in which subscript *M* denotes the quantity which will take the coordinate value of point *M*. Based on the above relations, Δ*_y_* may be computed as

(25) $$\Delta_{y}=AB=AC-BC=MC-BC=MC(1-\cos\theta )\approx MC\;\cdot \frac{1}{2}\theta^{2}.$$

Also noting that *MC = w_M_*/sin*θ= w_M_/(əw/əy)_M_*, by substituting it into Equation (25), we have

(26) $$\Delta_{y}=\frac{w_{M}}{{\left(\partial w/\partial y\right)}_{M}}\frac{1}{2}\theta^{2}=\frac{w_{M}}{{\left(\partial w/\partial y\right)}_{M}}\frac{1}{2}{\left(\frac{\partial w}{\partial y}\right)}_{M}^{2}=\frac{1}{2}w_{M}{\left(\frac{\partial w}{\partial y}\right)}_{M}.$$

Lastly, *W* may be computed as

(27) $$W=\frac{1}{2}\int_{0}^{l}qw_{M}{\left(\frac{\partial w}{\partial y}\right)}_{M}dx.$$

## 4. Application of Variational Method

In this section, we will derive the critical loads for the four stability problems with bimodular effect shown in Figure 2.

The whole procedure may be described as follows:

1. The first step is to select a deflection function with two unknown parameters. The deflection function should satisfy all boundary conditions of displacement.
2. Substituting the deflection function into Equations (16) and (27), the potential energy of strain, *U*, and the potential energy of external forces, *W*, may be computed.
3. Using Equations (1) and (2), the total potential energy of the system and its variation may be determined, resulting in two homogeneous linear equations with respect to the previously selected two parameters.
4. Letting the two linear equations have a nonzero solution, the coefficient determinant of the two equations must be zero; thus, another quadratic equation with respect to *q* may be obtained;
5. Finally, by solving the quadratic equation of *q*, its minimum real root will give the formulas of the corresponding critical load.

### 4.1. Bimodular Rectangular Plate with Top and Bottom Loaded

**Figure 5:**
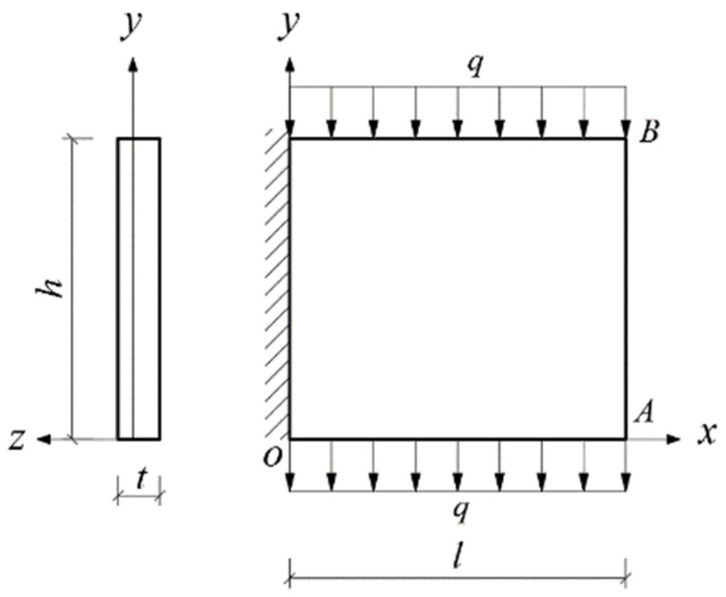
Bimodular rectangular plate with top and bottom loaded.

In Figure 5, the length, height and thickness of the plate are *l*, *h* and *t*, respectively; points *A* and *B* stand for the bottom and top corners of the right free side of the plate. Note that, as indicated in Section 3.1, due to the introduction of the bimodular effect, the neutral layer of the plate under bending is not located at the geometrical middle plane, and thus, the *y* axis deviates from the middle plane, as shown in Figure 5.

The following deflection function is selected [19]

(28) $$w=\left[f_{1}+(f_{2}-f_{1})\frac{y}{h}\right]\left(1-\cos\frac{\pi x}{2l}\right),$$

where *f*_1_ and *f*_2_ are the *z*-direction deflection of points *A* and *B* of the plate, respectively, and are unknown parameters. Note that a difference remains between Equation (28) in this study and the deflection function in [19] due to the different establishment modes of the coordinate system. From Equation (28), it is easy to see that when *x = 0*, we have *w* = 0 and ∂*w*/∂*x* = 0, which satisfies the boundary conditions of the entire fixed side; at the same time, when *x = l*, we have *w* = *f*_1_ + (*f*_2_ − *f*_1_*) y/h*, which also satisfies the displacement conditions of corner points *A* and *B*, that is, for point *A*, when *y* = *0*, we have *w* = *f*_1_. For point *B*, when *y* = *h*, *w* = *f*_2_ may be obtained. Therefore, Equation (28) is rational and may be used to obtain the solution.

Substituting Equation (28) into Equation (16) and integrating with respect to *y*, 0 to *h*, and with respect to *x*, 0 to *l*, we have

(29) $$\begin{array}{cc} U & =\frac{D^{+}\pi^{2}}{192l^{3}h}\left[\pi^{2}h^{2}(f_{1}^{2}+f_{2}^{2}+f_{1}f_{2})+24l^{2}(1-\mu^{+}){\left(f_{1}-f_{2}\right)}^{2}\right]\\ & +\frac{D^{-}\pi^{2}}{192l^{3}h}\left[\pi^{2}h^{2}(f_{1}^{2}+f_{2}^{2}+f_{1}f_{2})+24l^{2}(1-\mu^{-}){\left(f_{1}-f_{2}\right)}^{2}\right] \end{array}.$$

The work done by the external loads is divided into two parts: one is the uniformly-distributed load acting on the top of the plate, and the other is that acting on the bottom of the plate. For the work done by the former load, *W_t_*, by substituting Equation (28) into Equation (27), we obtain

(30) $$W_{t}=\frac{1}{2}{\int_{0}^{l}q\left(\frac{\partial w}{\partial y}w\right)}_{y=h}dx=\frac{(3\pi -8)ql}{4\pi h}f_{2}(f_{2}-f_{1}).$$

For the work done by the bottom load, *W_b_*,

(31) $$W_{b}=\frac{1}{2}{\int_{0}^{l}q\left(\frac{\partial w}{\partial y}w\right)}_{y=0}dx=\frac{(3\pi -8)ql}{4\pi h}f_{1}(f_{2}-f_{1}).$$

The total work done by the external loads is

(32) $$W=W_{t}+W_{b}=\frac{(3\pi -8)ql}{4\pi h}(f_{1}+f_{2})(f_{2}-f_{1}).$$

Substituting Equations (29) and (32) into Equation (1), we determine the total potential energy of the system

(33) $$\begin{array}{ll} \prod =U-W & =\frac{(3\pi -8)ql}{4\pi h}(f_{1}+f_{2})(f_{1}-f_{2})\\ & +\frac{D^{+}\pi^{2}}{192l^{3}h}\left[\pi^{2}h^{2}(f_{1}^{2}+f_{2}^{2}+f_{1}f_{2})+24l^{2}(1-\mu^{+}){\left(f_{1}-f_{2}\right)}^{2}\right]\\ & +\frac{D^{-}\pi^{2}}{192l^{3}h}\left[\pi^{2}h^{2}(f_{1}^{2}+f_{2}^{2}+f_{1}f_{2})+24l^{2}(1-\mu^{-}){\left(f_{1}-f_{2}\right)}^{2}\right] \end{array}$$

Variations of the two parameters *f*_1_ and *f*_2_ give the following two homogeneous linear equations for *f*_1_ and *f*_2_

(34) $$\begin{array}{ll} & \left[\frac{D^{+}\pi^{2}(\pi^{2}h^{2}+24l^{2}-24\mu^{+}l^{2})}{48l^{3}h}+\frac{D^{-}\pi^{2}(\pi^{2}h^{2}+24l^{2}-24\mu^{-}l^{2})}{48l^{3}h}+\frac{(3\pi -8)ql}{\pi h}\right]f_{1}\\ + & \left[\frac{D^{+}\pi^{2}(\pi^{2}h^{2}-48l^{2}+48\mu^{+}l^{2})}{96l^{3}h}+\frac{D^{-}\pi^{2}(\pi^{2}h^{2}-48l^{2}+48\mu^{-}l^{2})}{96l^{3}h}\right]f_{2}=0 \end{array}$$

and

(35) $$\begin{array}{l} \left[\frac{D^{+}\pi^{2}(\pi^{2}h^{2}-48l^{2}+48\mu^{+}l^{2})}{96l^{3}h}+\frac{D^{-}\pi^{2}(\pi^{2}h^{2}-48l^{2}+48\mu^{-}l^{2})}{96l^{3}h}\right]f_{1}+\\ \left[\frac{D^{+}\pi^{2}(\pi^{2}h^{2}+24l^{2}-24\mu^{+}l^{2})}{48l^{3}h}+\frac{D^{-}\pi^{2}(\pi^{2}h^{2}+24l^{2}-24\mu^{-}l^{2})}{48l^{3}h}-\frac{(3\pi -8)ql}{\pi h}\right]f_{2}=0 \end{array}.$$

To allow the system of Equations (34) and (35) to yield a nonzero solution, the coefficient determinant must be zero. Thus, we have

(36) $$\frac{{\left(3\pi -8\right)}^{2}l^{2}}{\pi^{2}h^{2}}q^{2}-\frac{\pi^{6}{\left(D^{+}+D^{-}\right)}^{2}}{3072l^{6}}\left(\pi^{2}h^{2}+96l^{2}-96l^{2}\frac{D^{+}\mu^{+}+D^{-}\mu^{-}}{D^{+}+D^{-}}\right)=0,$$

which gives the minimum real root, that is, the critical load for a bimodular rectangular plate with its top and bottom loaded

(37) $$q_{cr1}=\frac{2\pi }{3\pi -8}\frac{\pi^{2}(D^{+}+D^{-})}{4l^{2}}\left[\frac{\sqrt{2}}{4}\pi \frac{h}{l}\sqrt{\frac{\pi^{2}}{96}\frac{h^{2}}{l^{2}}+1-\frac{D^{+}\mu^{+}+D^{-}\mu^{-}}{D^{+}+D^{-}}}\right].$$

### 4.2. Bimodular Rectangular Plate with Top Loaded

**Figure 6:**
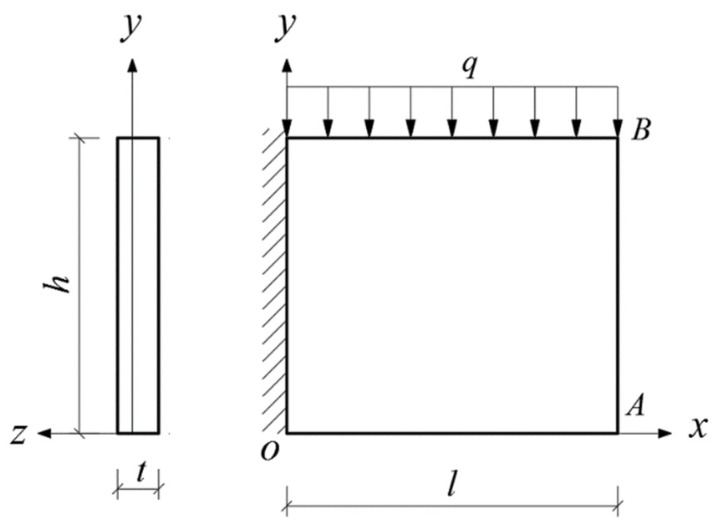
Bimodular rectangular plate with top loaded.

In this case, Equation (28) is still selected as the deflection function, in which the meanings of *f*_1_ and *f*_2_ remain unchanged, as shown in Figure 6. Note that here, the work done by the external load is *W_t_* in Equation (30). 

The total potential energy of the system is

(38) $$\begin{array}{ll} \prod =U-W_{t} & =\frac{(3\pi -8)ql}{4\pi h}f_{2}(f_{1}-f_{2})\\ & +\frac{D^{+}\pi^{2}}{192l^{3}h}\left[\pi^{2}h^{2}(f_{1}^{2}+f_{2}^{2}+f_{1}f_{2})+24l^{2}(1-\mu^{+}){\left(f_{1}-f_{2}\right)}^{2}\right]\\ & +\frac{D^{-}\pi^{2}}{192l^{3}h}\left[\pi^{2}h^{2}(f_{1}^{2}+f_{2}^{2}+f_{1}f_{2})+24l^{2}(1-\mu^{-}){\left(f_{1}-f_{2}\right)}^{2}\right] \end{array}$$

Similarly, the variations in *f*_1_ and *f*_2_ give the following two homogeneous linear equations for *f*_1_ and *f*_2_

(39) $$\begin{array}{l} \left[\frac{D^{+}\pi^{2}(\pi^{2}h^{2}+24l^{2}-24\mu^{+}l^{2})}{24l^{3}h}+\frac{D^{-}\pi^{2}(\pi^{2}h^{2}+24l^{2}-24\mu^{-}l^{2})}{24l^{3}h}\right]f_{1}+\\ \left[\frac{D^{+}\pi^{2}(\pi^{2}h^{2}+48l^{2}-48\mu^{+}l^{2})}{48l^{3}h}+\frac{D^{-}\pi^{2}(\pi^{2}h^{2}+48l^{2}-48\mu^{-}l^{2})}{48l^{3}h}+\frac{(3\pi -8)ql}{\pi h}\right]f_{2}=0 \end{array}$$

and

(40) $$\begin{array}{l} \left[\frac{D^{+}\pi^{2}(\pi^{2}h^{2}-48l^{2}+48\mu^{+}l^{2})}{96l^{3}h}+\frac{D^{-}\pi^{2}(\pi^{2}h^{2}-48l^{2}+48\mu^{-}l^{2})}{96l^{3}h}+\frac{(3\pi -8)ql}{2\pi h}\right]f_{1}+\\ \left[\frac{D^{+}\pi^{2}(\pi^{2}h^{2}+24l^{2}-24\mu^{+}l^{2})}{48l^{3}h}+\frac{D^{-}\pi^{2}(\pi^{2}h^{2}+24l^{2}-24\mu^{-}l^{2})}{48l^{3}h}-\frac{(3\pi -8)ql}{\pi h}\right]f_{2}=0 \end{array}.$$

The coefficient determinant of Equations (39) and (40) must be zero; thus

(41) $$\begin{array}{l} \frac{{\left(3\pi -8\right)}^{2}l^{2}}{\pi^{2}h^{2}}q^{2}+\frac{\pi^{3}(3\pi -8)(D^{+}+D^{-})}{8l^{2}}q\\ -\frac{\pi^{6}{\left(D^{+}+D^{-}\right)}^{2}}{768l^{6}}\left(\pi^{2}h^{2}+96l^{2}-96l^{2}\frac{D^{+}\mu^{+}+D^{-}\mu^{-}}{D^{+}+D^{-}}\right)=0 \end{array},$$

which will give the critical load for a bimodular rectangular plate with its top loaded, as follows

(42) $$q_{cr2}=\frac{2\pi }{3\pi -8}\frac{\pi^{2}(D^{+}+D^{-})}{4l^{2}}[-\frac{\pi^{2}}{8}\frac{h^{2}}{l^{2}}+\frac{\sqrt{2}}{2}\pi \frac{h}{l}\sqrt{\frac{\pi^{2}}{24}\frac{h^{2}}{l^{2}}+1-\frac{D^{+}\mu^{+}+D^{-}\mu^{-}}{D^{+}+D^{-}}}].$$

### 4.3. Bimodular Rectangular Plate with Bottom Loaded

**Figure 7:**
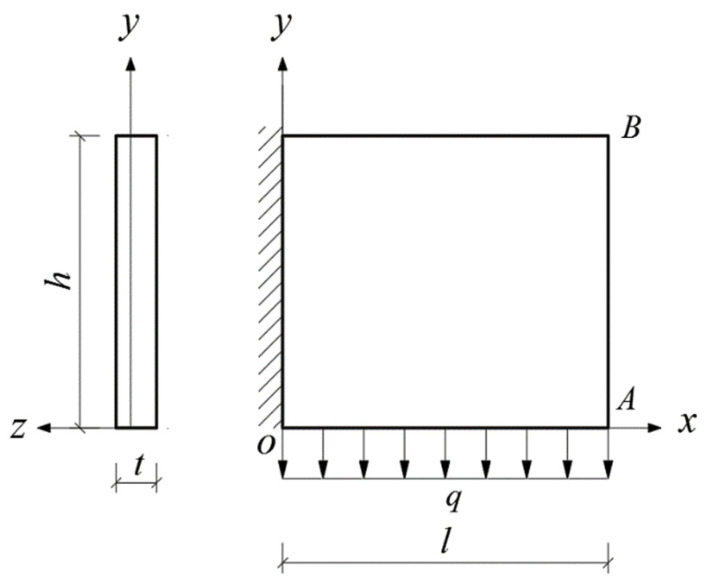
Bimodular rectangular plate with bottom loaded.

Equation (28) may still be used, and all other parameters remain the same as those shown in Figure 7. Note that the work done by the external load is *W_b_* in Equation (31); thus, we have

(43) $$\begin{array}{ll} \prod =U-W_{b} & =\frac{(3\pi -8)ql}{4\pi h}f_{1}(f_{1}-f_{2})\\ & +\frac{D^{+}\pi^{2}}{192l^{3}h}\left[\pi^{2}h^{2}(f_{1}^{2}+f_{2}^{2}+f_{1}f_{2})+24l^{2}(1-\mu^{+}){\left(f_{1}-f_{2}\right)}^{2}\right]\\ & +\frac{D^{-}\pi^{2}}{192l^{3}h}\left[\pi^{2}h^{2}(f_{1}^{2}+f_{2}^{2}+f_{1}f_{2})+24l^{2}(1-\mu^{-}){\left(f_{1}-f_{2}\right)}^{2}\right] \end{array}.$$

Similarly, the variations of *f*_1_ and *f*_2_ give the following two homogeneous linear equations for *f*_1_ and *f*_2_:

(44) $$\begin{array}{l} \left[\frac{D^{+}\pi^{2}(\pi^{2}h^{2}+24l^{2}-24\mu^{+}l^{2})}{48l^{3}h}+\frac{D^{-}\pi^{2}(\pi^{2}h^{2}+24l^{2}-24\mu^{-}l^{2})}{48l^{3}h}+\frac{(3\pi -8)ql}{\pi h}\right]f_{1}+\\ \left[\frac{D^{+}\pi^{2}(\pi^{2}h^{2}-48l^{2}+48\mu^{+}l^{2})}{96l^{3}h}+\frac{D^{-}\pi^{2}(\pi^{2}h^{2}-48l^{2}+48\mu^{-}l^{2})}{96l^{3}h}-\frac{(3\pi -8)ql}{2\pi h}\right]f_{2}=0 \end{array}$$

and

(45) $$\begin{array}{l} \left[\frac{D^{+}\pi^{2}(\pi^{2}h^{2}-48l^{2}+48\mu^{+}l^{2})}{48l^{3}h}+\frac{D^{-}\pi^{2}(\pi^{2}h^{2}-48l^{2}+48\mu^{-}l^{2})}{48l^{3}h}-\frac{(3\pi -8)ql}{\pi h}\right]f_{1}+\\ \left[\frac{D^{+}\pi^{2}(\pi^{2}h^{2}+24l^{2}-24\mu^{+}l^{2})}{24l^{3}h}+\frac{D^{-}\pi^{2}(\pi^{2}h^{2}+24l^{2}-24\mu^{-}l^{2})}{24l^{3}h}\right]f_{2}=0 \end{array}.$$

The coefficient determinant of Equations (44) and (45) must be zero; thus

(46) $$\begin{array}{l} \frac{{\left(3\pi -8\right)}^{2}l^{2}}{\pi^{2}h^{2}}q^{2}-\frac{\pi^{3}(3\pi -8)(D^{+}+D^{-})}{8l^{2}}q\\ -\frac{\pi^{6}{\left(D^{+}+D^{-}\right)}^{2}}{768l^{6}}\left(\pi^{2}h^{2}+96l^{2}-96l^{2}\frac{D^{+}\mu^{+}+D^{-}\mu^{-}}{D^{+}+D^{-}}\right)=0 \end{array},$$

which will give the critical load for a bimodular rectangular plate with its bottom loaded as follows:

(47) $$q_{cr3}=\frac{2\pi }{3\pi -8}\frac{\pi^{2}(D^{+}+D^{-})}{4l^{2}}[\frac{\pi^{2}}{8}\frac{h^{2}}{l^{2}}+\frac{\sqrt{2}}{2}\pi \frac{h}{l}\sqrt{\frac{\pi^{2}}{24}\frac{h^{2}}{l^{2}}+1-\frac{D^{+}\mu^{+}+D^{-}\mu^{-}}{D^{+}+D^{-}}}].$$

### 4.4. Bimodular Triangular Plate with Bottom Loaded

**Figure 8:**
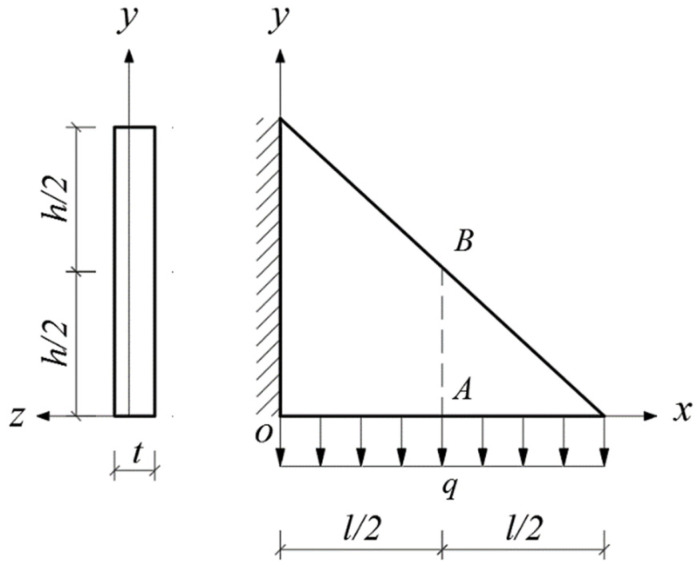
Bimodular triangular plate with bottom loaded.

In this case, the following deflection function is selected

(48) $$w=\left[f_{1}+(f_{2}-f_{1})\frac{2y}{h}\right]\left(1-\cos\frac{\pi x}{l}\right),$$

where *f*_1_ and *f*_2_ are the deflection of middle point *A* of the right angular edge and middle point *B* at oblique edge of the plate, respectively, as shown in Figure 8. Other quantities are the same as those for the rectangular plate. Note that there is a slight difference between Equation (48) and Equation (28) due to the change of the locations of points *A* and *B*. It is easy to verify that Equation (48) also satisfies all boundary conditions of displacement. Specifically, when *x =* 0, we have *w* = 0 and ∂*w*/∂*x* = 0, which satisfies the boundary conditions of the whole fixed side. When *x = l*, we have *w* = *f*_1_ + *2y/h* (*f*_2_ − *f*_1_*)*; this also satisfies the displacement conditions of points *A* and *B*, that is, for point *A*, when *y* = 0, *w* = *f*_1_, and for point *B*, when *y* = *h*/2, *w* = *f*_2_.

Substituting Equation (48) into Equation (16) (noting that the integration area is triangular), and thus integrating with respect to *y*, 0 to *h*, and with respect to *x*, 0 to *l*(1 − *y/h)*, we have

(49) $$\begin{array}{ll} U & =\frac{D^{+}}{2}\int_{0}^{h}dy\int_{0}^{l(1-\frac{y}{h})}\left[{\left(\frac{\partial^{2}w}{\partial x^{2}}\right)}^{2}+2(1-\mu^{+}){\left(\frac{\partial^{2}w}{\partial x\partial y}\right)}^{2}\right]dx\\ & +\frac{D^{-}}{2}\int_{0}^{h}dy\int_{0}^{l(1-\frac{y}{h})}\left[{\left(\frac{\partial^{2}w}{\partial x^{2}}\right)}^{2}+2(1-\mu^{-}){\left(\frac{\partial^{2}w}{\partial x\partial y}\right)}^{2}\right]dx\\ & =\frac{D^{+}\pi^{2}}{24hl^{3}}\left[\pi^{2}h^{2}(f_{1}^{2}+2f_{2}^{2})+24l^{2}(1-\mu^{+}){\left(f_{1}-f_{2}\right)}^{2}-6h^{2}(f_{1}f_{2}-f_{2}^{2})\right]\\ & +\frac{D^{-}\pi^{2}}{24hl^{3}}\left[\pi^{2}h^{2}(f_{1}^{2}+2f_{2}^{2})+24l^{2}(1-\mu^{-}){\left(f_{1}-f_{2}\right)}^{2}-6h^{2}(f_{1}f_{2}-f_{2}^{2})\right] \end{array}.$$

Substituting Equation (48) into Equation (27), the work done by the external load is

(50) $$W=\frac{3ql}{2h}f_{1}(f_{2}-f_{1}).$$

Substituting Equations (49) and (50) into Equation (1), the total potential energy of the system is

(51) $$\begin{array}{ll} \prod & =U-W\\ & =\frac{D^{+}\pi^{2}}{24hl^{3}}\left[\pi^{2}h^{2}(f_{1}^{2}+2f_{2}^{2})+24l^{2}(1-\mu^{+}){\left(f_{1}-f_{2}\right)}^{2}-6h^{2}(f_{1}f_{2}-f_{2}^{2})\right]\\ & +\frac{D^{-}\pi^{2}}{24hl^{3}}\left[\pi^{2}h^{2}(f_{1}^{2}+2f_{2}^{2})+24l^{2}(1-\mu^{-}){\left(f_{1}-f_{2}\right)}^{2}-6h^{2}(f_{1}f_{2}-f_{2}^{2})\right]\\ & -\frac{3ql}{2h}f_{1}(f_{2}-f_{1}) \end{array}.$$

The variations of *f*_1_ and *f*_2_ give the following two homogeneous linear equations for *f*_1_ and *f*_2_

(52) $$\begin{array}{l} \left[\frac{D^{+}\pi^{2}(\pi^{2}h^{2}+24l^{2}-24\mu^{+}l^{2})}{12l^{3}h}+\frac{D^{-}\pi^{2}(\pi^{2}h^{2}+24l^{2}-24\mu^{-}l^{2})}{12l^{3}h}+\frac{3ql}{h}\right]f_{1}+\\ \left[\frac{D^{+}\pi^{2}(-h^{2}-8l^{2}+8\mu^{+}l^{2})}{4l^{3}h}+\frac{D^{-}\pi^{2}(-h^{2}-8l^{2}+8\mu^{-}l^{2}}{4l^{3}h})-\frac{3ql}{2h}\right]f_{2}=0 \end{array}$$

and

(53) $$\begin{array}{l} \left[\frac{D^{+}\pi^{2}(-h^{2}-8l^{2}+8\mu^{+}l^{2})}{2l^{3}h}+\frac{D^{-}\pi^{2}(-h^{2}-8l^{2}+8\mu^{+}l^{2})}{2l^{3}h}-\frac{3ql}{h}\right]f_{1}+\\ \left[\frac{D^{+}\pi^{2}(\pi^{2}h^{2}+3h^{2}+12l^{2}-12\mu^{+}l^{2})}{3l^{3}h}+\frac{D^{-}\pi^{2}(\pi^{2}h^{2}+3h^{2}+12l^{2}-12\mu^{+}l^{2})}{3l^{3}h}\right]f_{2}=0 \end{array}.$$

The coefficient determinant of Equations (52) and (53) must be zero; thus

(54) $$\begin{array}{l} \frac{9l^{2}}{h^{2}}q^{2}-\frac{\pi^{2}(2\pi^{2}+3)(D^{+}+D^{-})}{l^{2}}q\\ -\frac{\pi^{4}{\left(D^{+}+D^{-}\right)}^{2}}{36l^{6}}\left[h^{2}(2\pi^{4}+6\pi^{2}-9)+72\pi^{2}l^{2}\left(1-\frac{D^{+}\mu^{+}+D^{-}\mu^{-}}{D^{+}+D^{-}}\right)\right]=0 \end{array},$$

giving the critical load for a bimodular triangular plate with its bottom loaded, as follows

(55) $$q_{cr4}=\frac{\pi^{2}(D^{+}+D^{-})}{4l^{2}}[\left(\frac{4\pi^{2}}{9}+\frac{2}{3}\right)\frac{h^{2}}{l^{2}}+\frac{4\sqrt{2}}{3}\pi \frac{h}{l}\sqrt{\left(\frac{\pi^{2}}{12}+\frac{1}{4}\right)\frac{h^{2}}{l^{2}}+1-\frac{D^{+}\mu^{+}+D^{-}\mu^{-}}{D^{+}+D^{-}}}].$$

## 5. Results and Discussions

### 5.1. Comparision of Four Critical Loads without Bimodular Effect

First, let us compare the critical loads of four stability problems without the bimodular effect, which are denoted by *q_cr_*_1_*, *q_cr_*_2_*, *q_cr_*_3_* and *q_cr_*_4_*. For this purpose, let *D^+^* = *D*^−^ = *D*/2 and *μ^+^* = *μ*^−^ = *μ* in Equations (37), (42), (47) and (55). Thus, for a rectangular plate with its top and bottom loaded, we have

(56) $$q_{cr1}{}^{*}=\frac{2\pi }{3\pi -8}\frac{\pi^{2}D}{4l^{2}}\left(\frac{\sqrt{2}}{4}\pi \frac{h}{l}\sqrt{\frac{\pi^{2}}{96}\frac{h^{2}}{l^{2}}+1-\mu }\right).$$

For the rectangular plate with its top loaded, we have

(57) $$q_{cr2}{}^{*}=\frac{2\pi }{3\pi -8}\frac{\pi^{2}D}{4l^{2}}\left(-\frac{\pi^{2}}{8}\frac{h^{2}}{l^{2}}+\frac{\sqrt{2}}{2}\pi \frac{h}{l}\sqrt{\frac{\pi^{2}}{24}\frac{h^{2}}{l^{2}}+1-\mu }\right).$$

For the rectangular plate with its bottom loaded, we have

(58) $$q_{cr3}{}^{*}=\frac{2\pi }{3\pi -8}\frac{\pi^{2}D}{4l^{2}}\left(\frac{\pi^{2}}{8}\frac{h^{2}}{l^{2}}+\frac{\sqrt{2}}{2}\pi \frac{h}{l}\sqrt{\frac{\pi^{2}}{24}\frac{h^{2}}{l^{2}}+1-\mu }\right).$$

For the triangular plate with its bottom loaded, we have

(59) $$q_{cr4}{}^{*}=\frac{\pi^{2}D}{4l^{2}}[\left(\frac{4\pi^{2}}{9}+\frac{2}{3}\right)\frac{h^{2}}{l^{2}}+\frac{4\sqrt{2}}{3}\pi \frac{h}{l}\sqrt{\left(\frac{\pi^{2}}{12}+\frac{1}{4}\right)\frac{h^{2}}{l^{2}}+1-\mu }].$$

**Figure 9:**
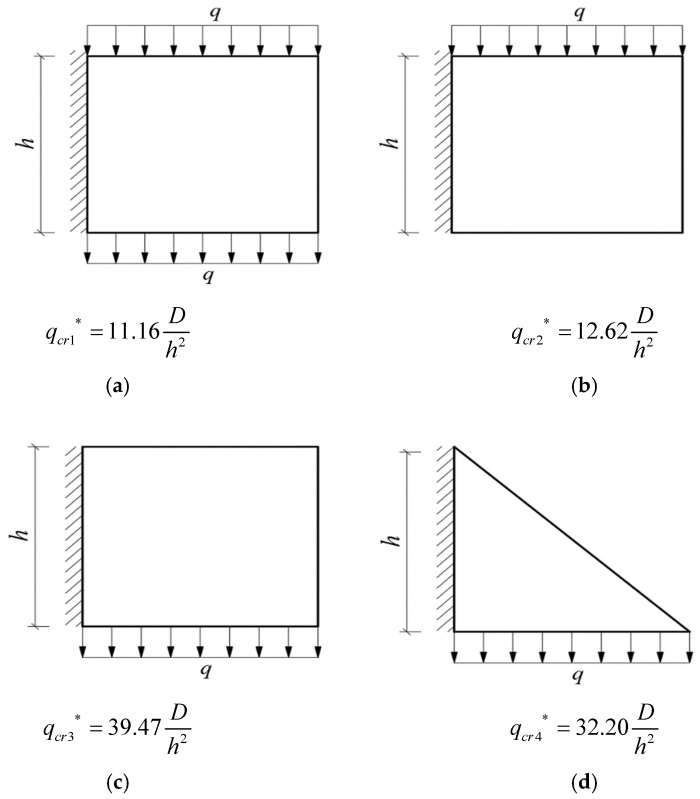
Four stability problems and their corresponding critical loads without bimodular effect. (**a**) Rectangular plate with its top and bottom loaded; (**b**) Rectangular plate with its top loaded; (**c**) Rectangular plate with its bottom loaded; and (**d**) Triangular plate with its bottom loaded.

For the sake of comparison, we take *μ* = 0.25 and *l* = *h*, thus obtaining the following figure (see Figure 9) in which there is no need to describe the coordinate system, as the solution depends only on *D* and *h.*

Due to the fact that the magnitude of the critical load is closely related to tendency of the plate to buckle, the following conclusions may be drawn from Figure 9: (1) Among the four cases, the order is, by the degree of ease of buckling, *q_cr_*_1_* < *q_cr_*_2_* < *q_cr_*_4_* < *q_cr_*_3_*; (2) For the rectangular plates, case (a) with top and bottom loaded is most likely to buckle, followed by case (b) with top loaded, and then case (c) with bottom loaded; (3) By comparing (c) with (d), it is easy to see that, under the same load, the triangular plate is more prone to buckling than the rectangular plate.

Cheng [19] also determined the critical load of a cantilever vertical plate with its top loaded without considering the bimodular effect. His solution was as follows

(60) $${\left(q_{0}\right)}_{cr}=\frac{2\pi }{3\pi -8}\frac{\pi^{2}D}{4b^{2}}\left[-\frac{\pi^{2}}{8}\frac{a^{2}}{b^{2}}+\frac{\sqrt{2}}{2}\pi \frac{a}{b}{\left(\frac{\pi^{2}}{24}\frac{a^{2}}{b^{2}}+1-\mu \right)}^{1/2}\right],$$

where (*q*_0_)*_cr_* is the critical load, *a* and *b* are the height and length of the cantilever vertical plate, respectively, and *D* is the bending stiffness of the plate. Equation (57) in this study and Equation (60) in [19] are the same. This verifies, to some extent, the correctness of the analytical solutions derived in this study; however, in our study, the bimodular effect is introduced and other loading types and plate shapes are also considered. Of course, a demonstration of validity should include a numerical simulation based on FEM, in which a real material model may be incorporated. Nonetheless, as indicated in the Introduction, for a bimodular problem, it is difficult and time-consuming to use the finite element method (FEM) based on an iterative technique. In addition, there is no bimodular materials model in existing commercial FEM software, e.g., ABAQUS. New material types may be introduced through the use of external modules, for example, by applying the user materials subroutine, UMAT, to evaluate the mechanical behavior of a given type of material. Given that the main aim of this study is to use the variation method to determine the critical loads of various components, more accurate numerical simulations will be considered in future.

### 5.2. Bimodular Effect on Four Critical Loads

For the investigation of the bimodular effect on the critical loads, let us introduce the following relations [39]

(61) $$\begin{array}{llll} E=\frac{E^{+}+E^{-}}{2}, & \;\;\beta =\frac{E^{+}-E^{-}}{E^{+}+E^{-}}, & \;\;E^{+}=(1+\beta )E, & \;E^{-}=(1-\beta )E\\ \mu =\frac{\mu^{+}+\mu^{-}}{2}, & \;\beta =\frac{\mu^{+}-\mu^{-}}{\mu^{+}+\mu^{-}}, & \;\mu^{+}=(1+\beta )\mu , & \;\mu^{-}=(1-\beta )\mu \end{array},$$

where *E* is the average value of the tensile and compressive moduli, and *μ* is the average value of the tensile and compressive Poisson ratio. *β* is an important parameter which may be positive or negative. In addition, we introduce the following dimensionless quantities

(62) $$\begin{array}{l} T_{1}=\frac{t_{1}}{t},T_{2}=\frac{t_{2}}{t},\lambda_{h}=\frac{h}{l},\lambda_{t}=\frac{t}{l},Q_{cr}=\frac{q_{cr}}{Et}\\ K^{+}=\frac{D^{+}}{Et^{3}}=\frac{(1+\beta )T_{1}^{3}}{3[1-{\left(\mu^{+}\right)}^{2}]},K^{-}=\frac{D^{-}}{Et^{3}}=\frac{(1-\beta )T_{2}^{3}}{3[1-{\left(\mu^{-}\right)}^{2}]} \end{array}.$$

Using the relations in Equations (61) and (62), we may obtain the dimensionless forms of Equations (37), (42), (47) and (55). For the rectangular plate with its top and bottom loaded, we have

(63) $$Q_{cr1}=\frac{2\pi }{3\pi -8}\frac{\pi^{2}\lambda_{t}^{2}(K^{+}+K^{-})}{4}\left(\frac{\sqrt{2}}{4}\pi \lambda_{h}\sqrt{\frac{\pi^{2}}{96}\lambda_{h}^{2}+1-\frac{K^{+}\mu^{+}+K^{-}\mu^{-}}{K^{+}+K^{-}}}\right).$$

For the rectangular plate with its top loaded, we have

(64) $$Q_{cr2}=\frac{2\pi }{3\pi -8}\frac{\pi^{2}\lambda_{t}^{2}(K^{+}+K^{-})}{4}\left(-\frac{\pi^{2}}{8}\lambda_{h}^{2}+\frac{\sqrt{2}}{2}\pi \lambda_{h}\sqrt{\frac{\pi^{2}}{24}\lambda_{h}^{2}+1-\frac{K^{+}\mu^{+}+K^{-}\mu^{-}}{K^{+}+K^{-}}}\right).$$

For the rectangular plate with its bottom loaded, we have

(65) $$Q_{cr3}=\frac{2\pi }{3\pi -8}\frac{\pi^{2}\lambda_{t}^{2}(K^{+}+K^{-})}{4}\left(\frac{\pi^{2}}{8}\lambda_{h}^{2}+\frac{\sqrt{2}}{2}\pi \lambda_{h}\sqrt{\frac{\pi^{2}}{24}\lambda_{h}^{2}+1-\frac{K^{+}\mu^{+}+K^{-}\mu^{-}}{K^{+}+K^{-}}}\right).$$

For the triangular plate with its bottom loaded, we have

(66) $$Q_{cr4}=\frac{\pi^{2}\lambda_{t}^{2}(K^{+}+K^{-})}{4}[\left(\frac{4\pi^{2}}{9}+\frac{2}{3}\right)\lambda_{h}^{2}+\frac{4\sqrt{2}}{3}\pi \lambda_{h}\sqrt{\left(\frac{\pi^{2}}{12}+\frac{1}{4}\right)\lambda_{h}^{2}+1-\frac{K^{+}\mu^{+}+K^{-}\mu^{-}}{K^{+}+K^{-}}}].$$

Up to now, we have determined the dimensionless critical loads which may be numerical determined via known parameters such as *λ_h_*, *λ_t_*, *μ^+^*, *μ*^−^, *K*^+^ and *K*^−^, in which the *T_1_* and *T_2_* contained in *K*^+^ and *K*^−^ are, based on Equation (23)

(67) $$T_{1}=\frac{\sqrt{\left(1-\beta )(1-\mu^{+}\right)}}{\sqrt{\left(1+\beta )(1-\mu^{-}\right)}+\sqrt{\left(1-\beta )(1-\mu^{+}\right)}}{,}_{}^{}T_{2}=\frac{\sqrt{\left(1+\beta )(1-\mu^{-}\right)}}{\sqrt{\left(1+\beta )(1-\mu^{-}\right)}+\sqrt{\left(1-\beta )(1-\mu^{+}\right)}}.$$

Table 1 lists the value of *β* taken from Equation (61), ranging from 0.3 to −0.3, with an interval of 0.05. Thus, there are, in total, thirteen data groups. Table 1 also lists the corresponding computational value of related physical quantities including *μ^+^* and *μ*^−^ from Equation (61), in which the average modulus, *μ*, is first set to 0.25, and also includes *T*_1_ and *T*_2_ from Equation (67), as well as *K*^+^ and *K*^−^ from Equation (62). *β* = 0 indicates that the tensile modulus is equal to the compressive one, and also that, in this case, we have *μ^+^* = *μ*^−^, *T*_1_ = *T*_2_ and *K*^+^ = *K*^−^. From the data of Table 1, it is easy to observe a regular pattern, indicating that when the abstract values of *β* remain unchanged, only a change of positive or negative sign occurs for *μ^+^* and *μ*^−^, *T*_1_ and *T*_2_, and *K*^+^ and *K*^−^, which exchange their values. Specifically, if *β* changes from 0.05 to −0.05, *μ^+^* = 0.2625 when *β* = 0.05 is exactly *μ*^−^ = 0.2625 when *β* = −0.05, while at the same time, *μ*^−^ = 0.2375 when *β* = 0.05 is exactly *μ^+^* = 0.2375 when *β* = −0.05. A similar pattern may be found for *T*_1_ and *T*_2_ and *K*^+^ and *K*^−^. This fact reminds us that if these quantities are symmetrically distributed in the expressions for the critical load (the so-called symmetry may be found in Equations (61) to (66)), as long as the abstract value of *β* remains unchanged, whether this value is positive or negative, the final calculated value of the critical loads remains unchanged, that is, the relation of *Q_cri_* (*i* = 1,2,3,4) and *β* satisfies

(68) $$Q_{cri}(-\beta )=Q_{cri}(\beta ),(i=1,2,3,4).$$

It is easy to see from Table 1 that the dimensionless bending stiffness, *K*= *K*^+^ + *K*^−^, also forms a regular pattern, that is

(69) K(−β)=K(β).

These results may be helpful to the following analysis and discussions.

Table 2, Table 3, Table 4, Table 5, Table 6, Table 7 and Table 8 list four critical loads under different *λ_h_* and *λ_t_* when *β* = 0, ±0.05, ±0.1, ±0.15, ±0.2, ±0.25 and ±0.3, and *λ_h_* ranges from 0.9 to 1.1 and *λ_t_* from 0.02 to 0.04, both according to real conditions in the cantilever vertical plates used for the design of balconies. Specifically, one designer may refer to the dimensionless value of critical loads to determine the real critical loads without much effort via the expression *q_cr_ = EtQ_cr_*, where *E* is the average modulus and *t* is the thickness of the plate.

From Table 2, Table 3, Table 4, Table 5, Table 6, Table 7 and Table 8, it is easy to find the influences of parameters *λ_h_* and *λ_t_* on the critical loads. As indicated in Equation (62), *λ_h_* = *h*/*l*, ranging from 0.9 to 1.1. When *λ_h_* is 0.9, 1.0 and 1.1, this corresponds to the cases of *h* < *l*, *h* = *l* (square plate) and *h* > *l*. It may be observed that, according to the magnitudes of the critical loads, *h* < *l* is most likely to buckle, followed by the square plate, while *h* > *l* is least likely to buckle. For the thickness variation of *λ_t_ = t/l*, it is obvious that the increase in thickness improves instability; thus, *λ_t_ =* 0.02 is most likely to buckle, followed by *λ_t_ =* 0.03, while *λ_t_ =* 0.04 is the least likely to buckle.

From Table 2, Table 3, Table 4, Table 5, Table 6, Table 7 and Table 8, we can find the basic tendency is still, according to the degree of buckling, *Q_cr_*_1_ > *Q_cr_*_2_ > *Q_cr_*_4_ > *Q_cr_*_3_, indicating that regardless of the values of *β*, *λ_h_* and *λ_t_*, a rectangular plate with its top and bottom loaded is most likely to buckle, followed by the rectangular plate only with its top loaded, the triangular plate with its bottom loaded, and finally, the rectangular plate only with its bottom loaded. The difference magnitudes among the critical loads of four groups are also different. From Table 2, Table 3, Table 4, Table 5, Table 6, Table 7 and Table 8, it is easy to see that there are smaller differences between *Q_cr_*_1_ and *Q_cr_*_2_, as well as slightly bigger differences between *Q_cr_*_3_ and *Q_cr_*_4_, while larger differences may be observed between *Q_cr_*_1_ (or *Q_cr_*_2_) and *Q_cr_*_3_ (or *Q_cr_*_4_).

**Figure 10:**
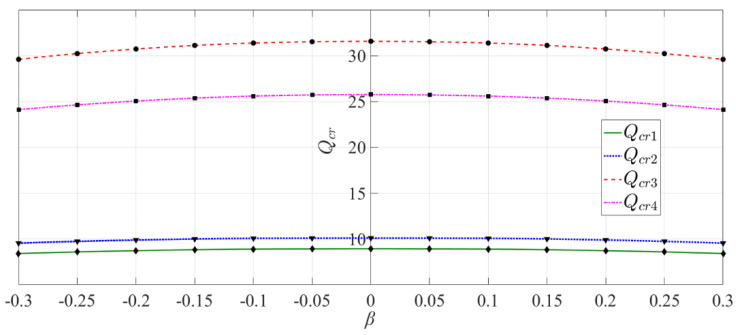
Variation of four critical loads *Q_cr_* with *β* (*λ_h_* = 1.0 and *λ_t_* = 0.03).

Figure 10 shows the variation curves of four critical loads with *β* values. Although it is plotted for a given case, for example, *λ_h_* = 1.0 and *λ_t_* = 0.03, it is applicable to other cases of values of *λ_h_* and *λ_t_*. It is easy to see from Figure 10 that *Q_cr_*_1_ > *Q_cr_*_2_ > *Q_cr_*_4_ > *Q_cr_*_3_, as well as the relative differences among the four critical loads, that is, *Q_cr_*_1_ is very close to *Q_cr_*_2_, *Q_cr_*_3_ is relatively close to *Q_cr_*_4_, but there is larger difference between *Q_cr_*_1_ (or *Q_cr_*_2_) and *Q_cr_*_3_ (or *Q_cr_*_4_). In addition, the two curves of *Q_cr_*_3_ and *Q_cr_*_4_ have obvious arch shapes, indicating that *β* has a greater influence on *Q_cr_*_3_ and *Q_cr_*_4_, while the two curves of *Q_cr_*_1_ and *Q_cr_*_2_ approach a flat line, indicating a smaller influence of *β* on *Q_cr_*_1_ and *Q_cr_*_2_. It may concluded therefore that the introduction of bimodular effect has a greater influence on rectangular and triangular cantilever vertical plates with their bottoms loaded, and a smaller influence on rectangular plates with either their top and bottom loaded or only the top loaded.

**Figure 11:**
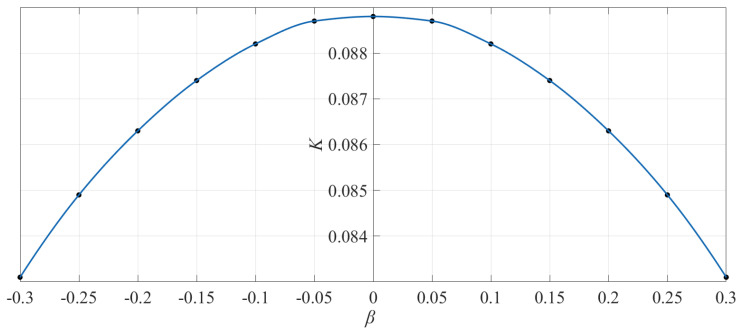
Variation of bending stiffness *K* with *β* (*λ_h_* = 1.0 and *λ_t_* = 0.03).

Figure 11 shows the variation of dimensionless bending stiffness *K* with *β*, suggesting the same arch shape as the critical loads *Q_cr_*, which indicates that the variation of *Q_cr_* with *β* is similar to that of *K* with *β*. From Equations (56)–(59), it is easy to see that q_cr_* is directly proportional to the bending stiffness *D* in a uniform modulus case, but what happens if the bimodular effect is introduced? From Equations (63)–(66), it is found that *Q_cr_* is not proportional to the bending stiffness *K* in a bimodular case; in contrast to Equations (56)–(59), the relation between *Q_cr_* and *K* seems to be nonlinear. However, by considering Equations (63)–(66), it may be seen that if we neglect the difference of *μ^+^* and *μ*^−^, and further take *μ^+^* = *μ*^−^ = *μ*, then the last item in the square root will become *μ*. Thus, the resulting *Q_cr_* is also proportional to the bending stiffness *K* in a bimodular problem. This conclusion is rational, since the influence of Poisson ratio *μ* is generally regarded as secondary, compared with the influence of the modulus of elasticity. It is for this reason that, at present, most studies do not take into account the influence of Poisson ratio on stress and deformation; of course, this also includes the critical loads discussed here.

## 6. Concluding Remarks

In this study, a variational method based on bimodular theory is applied to solve the stability problems of cantilever vertical plates used in balcony structures, in which different loading types and different plate shapes are considered. The results indicate that these factors influence the final critical loads. The following four conclusions can be drawn.

1. Among the four plate cases, the rectangular plate with its top and bottom loaded is most likely to buckle; next is the rectangular plate with its top loaded, followed by the triangular plate with its bottom loaded. The rectangular plate with its bottom loaded is least likely to buckle.
2. Among the four plate cases, the introduction of bimodular effect has a greater influence on rectangular and triangular plates with their bottoms loaded, but has a smaller influence on the rectangular plate with its top and bottom loaded, as well as on the rectangular plate with its top loaded.
3. If the average value of the tensile and compressive moduli, *E =* (*E*^+^ + *E*^−^)/2, remains unchanged, the introduction of bimodular effect, either *E*^+^ > *E*^−^, or *E*^+^ < *E*^−^, will weaken the bending stiffness of the plate compared with the original uniform modulus case. Specifically, the greater the difference between the tensile and compressive moduli, the smaller the resulting bending stiffness, and accordingly, the more likely that they will buckle.
4. If we neglect the influence of the Poisson ratio, the critical load in a bimodular problem is also directly proportional to the corresponding bending stiffness of the bimodular plate, which is the same as the classical problem with a uniform modulus.

This study may serve as a theoretical reference for the analysis and design of concrete vertical plates, especially those using special concretes whose bimodular effect is relatively significant and cannot be ignored arbitrarily. In addition, this study successfully applies the variational method to determine the critical loads of stability problems taking the bimodular effect into account. Although there was no substantial change to the method itself, its implementation requires knowledge of the bending strain energy based on bimodular theory, which may be seen as an extension of this method. Lastly, the problem and method proposed in this study may also be used for the analysis and design of other mechanical components associated with structural engineering.

## Figures and Tables

**Table 1:** Values of *β* and the corresponding computational values.

*β*	*μ* ^+^	*μ* ^−^	*T*_1_	*T*_2_	*K* ^+^	*K* ^−^
0.3	0.325	0.175	0.3989	0.6011	0.0308	0.0523
0.25	0.3125	0.1875	0.4161	0.5839	0.0333	0.0516
0.2	0.3	0.2	0.4330	0.5670	0.0357	0.0506
0.15	0.2875	0.2125	0.4499	0.5501	0.0380	0.0494
0.1	0.275	0.225	0.4666	0.5334	0.0403	0.0479
0.05	0.2625	0.2375	0.4833	0.5167	0.0424	0.0463
0	0.25	0.25	0.5	0.5	0.0444	0.0444
−0.05	0.2375	0.2625	0.5167	0.4833	0.0463	0.0424
−0.1	0.225	0.275	0.5334	0.4666	0.0479	0.0403
−0.15	0.2125	0.2875	0.5501	0.4499	0.0494	0.0380
−0.2	0.2	0.3	0.5670	0.4330	0.0506	0.0357
−0.25	0.1875	0.3125	0.5839	0.4161	0.0516	0.0333
−0.3	0.175	0.325	0.6011	0.3989	0.0523	0.0308

**Table 2:** Four critical loads under different *λ_h_* and *λ_t_* (***β =* 0**).

*λ* *_h_*	*λ* *_t_*	*Q_cr_*_1_(×10^−4^)	*Q_cr_*_2_(×10^−4^)	*Q_cr_*_3_(×10^−4^)	*Q_cr_*_4_(×10^−4^)
0.9	0.02	3.530	4.183	11.91	9.541
1.0	0.02	3.968	4.488	14.03	11.44
1.1	0.02	4.420	4.784	16.33	13.54
0.9	0.03	7.943	9.413	26.81	21.46
1.0	0.03	8.928	10.09	31.57	25.76
1.1	0.03	9.945	10.76	36.75	30.47
0.9	0.04	14.12	16.73	47.66	38.16
1.0	0.04	15.87	17.95	56.13	45.79
1.1	0.04	17.68	19.13	65.33	54.17

**Table 3:** Four critical loads under different *λ_h_* and *λ_t_* (***β =* ±0.05**).

*λ* *_h_*	*λ* *_t_*	*Q_cr_*_1_(×10^−4^)	*Q_cr_*_2_(×10^−4^)	*Q_cr_*_3_(×10^−4^)	*Q_cr_*_4_(×10^−4^)
0.9	0.02	3.524	4.176	11.89	9.524
1.0	0.02	3.961	4.480	14.00	11.42
1.1	0.02	4.412	4.775	16.30	13.52
0.9	0.03	7.929	9.396	26.76	21.43
1.0	0.03	8.912	10.08	31.52	25.71
1.1	0.03	9.927	10.74	36.68	30.42
0.9	0.04	14.09	16.70	47.57	38.09
1.0	0.04	15.84	17.92	56.03	45.71
1.1	0.04	17.64	19.10	65.22	54.08

**Table 4:** Four critical loads under different *λ_h_* and *λ_t_* (***β =* ±0.1**).

*λ* *_h_*	*λ* *_t_*	*Q_cr_*_1_(×10^−4^)	*Q_cr_*_2_(×10^−4^)	*Q_cr_*_3_(×10^−4^)	*Q_cr_*_4_(×10^−4^)
0.9	0.02	3.514	4.169	11.84	9.481
1.0	0.02	3.949	4.473	13.95	11.37
1.1	0.02	4.399	4.768	16.23	13.45
0.9	0.03	7.906	9.381	26.65	21.33
1.0	0.03	8.887	10.06	31.38	25.59
1.1	0.03	9.898	10.72	36.53	30.27
0.9	0.04	14.05	16.67	47.38	37.92
1.0	0.04	15.79	17.89	55.80	45.50
1.1	0.04	17.59	19.07	64.94	53.82

**Table 5:** Four critical loads under different *λ_h_* and *λ_t_* (***β =* ±0.15**).

*λ* *_h_*	*λ* *_t_*	*Q_cr_*_1_(×10^−4^)	*Q_cr_*_2_(×10^−4^)	*Q_cr_*_3_(×10^−4^)	*Q_cr_*_4_(×10^−4^)
0.9	0.02	3.486	4.139	11.74	9.398
1.0	0.02	3.918	4.441	13.83	11.27
1.1	0.02	4.364	4.733	16.09	13.33
0.9	0.03	7.845	9.314	26.43	21.14
1.0	0.03	8.817	9.992	31.12	25.37
1.1	0.03	9.820	10.65	36.21	30.01
0.9	0.04	13.94	16.55	46.98	37.59
1.0	0.04	15.67	17.76	55.33	45.10
1.1	0.04	17.45	18.93	64.38	53.35

**Table 6:** Four critical loads under different *λ_h_* and *λ_t_* (***β =* ±0.2**).

*λ* *_h_*	*λ* *_t_*	*Q_cr_*_1_(×10^−4^)	*Q_cr_*_2_(×10^−4^)	*Q_cr_*_3_(×10^−4^)	*Q_cr_*_4_(×10^−4^)
0.9	0.02	3.446	4.093	11.60	9.281
1.0	0.02	3.873	4.392	13.66	11.13
1.1	0.02	4.313	4.681	15.89	13.16
0.9	0.03	7.753	9.211	26.10	20.88
1.0	0.03	8.714	9.882	30.73	25.05
1.1	0.03	9.705	10.53	35.77	29.63
0.9	0.04	13.78	16.37	46.41	37.12
1.0	0.04	15.49	17.56	54.64	44.53
1.1	0.04	17.25	18.72	63.59	52.67

**Table 7:** Four critical loads under different *λ_h_* and *λ_t_* (***β =* ±0.25**).

*λ* *_h_*	*λ* *_t_*	*Q_cr_*_1_(×10^−4^)	*Q_cr_*_2_(×10^−4^)	*Q_cr_*_3_(×10^−4^)	*Q_cr_*_4_(×10^−4^)
0.9	0.02	3.391	4.031	11.41	9.126
1.0	0.02	3.811	4.325	13.43	10.94
1.1	0.02	4.245	4.610	15.63	12.94
0.9	0.03	7.631	9.071	25.67	20.53
1.0	0.03	8.576	9.732	30.23	24.63
1.1	0.03	9.551	10.37	35.18	29.13
0.9	0.04	13.56	16.12	45.65	36.50
1.0	0.04	15.24	17.30	53.75	43.79
1.1	0.04	16.98	18.44	62.54	51.79

**Table 8:** Four critical loads under different *λ_h_* and *λ_t_* (***β = *±0.3**).

*λ* *_h_*	*λ* *_t_*	*Q_cr_*_1_(×10^−4^)	*Q_cr_*_2_(×10^−4^)	*Q_cr_*_3_(×10^−4^)	*Q_cr_*_4_(×10^−4^)
0.9	0.02	3.323	3.952	11.17	8.935
1.0	0.02	3.734	4.240	13.15	10.71
1.1	0.02	4.159	4.519	15.30	12.67
0.9	0.03	7.477	8.894	25.14	20.10
1.0	0.03	8.403	9.541	29.60	24.11
1.1	0.03	9.358	10.16	34.44	28.52
0.9	0.04	13.29	15.81	44.70	35.74
1.0	0.04	14.93	16.96	52.63	42.87
1.1	0.04	16.63	18.07	61.23	50.70

## Data Availability

Not applicable.

## Nomenclature

*x*, *y*, *z*	Rectangular coordinates
*o*	Origin of the rectangular coordinate system (*x*, *y*, *z*)
*h*	Height of the cantilever vertical plate
*l*	Length of the cantilever vertical plate
*q*	Uniformly-distributed loads acting on the top or/and bottom of the cantilever vertical plate (see Figure 2)
*p*	Normal uniformly-distributed loads acting on the upper surface of the horizontal plate (see Figure 3)
*U*	Strain potential energy
*W*	Potential energy of external loads
*∏*	Total potential energy of the system
δ	Variation symbol
*E* ^+^	Tensile Young’s modulus of elasticity
*E* ^–^	Compressive Young’s modulus of elasticity
*μ*+	Tensile Poisson ratio
*μ* ^−^	Compressive Poisson ratio
*t*	Thickness of the cantilever vertical plate
*t*_1_	Tensile thickness of the plate
*t*_2_	Compressive thickness of the plate
*w*	Deflection of the plate
*ε_x_*, *ε_y_*, *ε_z_*	Normal strains in *x*, *y* and *z* directions
*γ_xy_*, *γ_yz_*, *γ_zx_*	Shearing strains in rectangular coordinates
*σ_x_*^+^, *σ_y_*^+^, *τ_xy_*^+^	Tensile stress components in two-dimensional thin plate theory
*σ_x_*^−^, *σ_y_*^−^, *τ_xy_*^−^	Compressive stress components in two-dimensional thin plate theory
*M_x_*, *M_y_*	Bending moments per unit length on the sections normal to *x*-axis and *y*-axis
*M_xy_*	Torsion moment per unit length on the section normal to *x*-axis
▽^4^	Biharmonic operator
*D*	Bending stiffness of a bimodular plate, *D* = *D*^+^ + *D*^−^
*D* ^+^	Tensile part of bending stiffness *D*
*D* ^−^	Compressive part of bending stiffness *D*
*N_x_*, *N_y_*	Normal forces per unit length on the sections normal to *x*-axis and *y*-axis
Δ*y*	Displacement of the action point of load q (see Figure 4)
*θ*	Rotation angle (see Figure 4)
*f*_1_, *f*_2_	*z*-direction deflection of bottom corner point *A* and top corner points *B* of the rectangular plate (see Figure 5, Figure 6 and Figure 7); *z*-direction deflection of middle point *A* of the right angular edge and middle point *B* at oblique edge of the triangular plate (see Figure 8)
*W_t_*	Work done by the top load
*W_b_*	Work done by the bottom load
*π*	Pi (the ratio of circumference to diameter)
*q*_*cr*1_*, *q*_*cr*2_*, *q*_*cr*3_*, *q*_*cr*4_*	Critical loads of four stability problems without bimodular effect (see Figure 9, in which *μ* = 0.25 and *l* = *h*)
*E*	Average quantity of *E*^+^ and *E*^−^
*μ*	Average quantity of *μ*+ and *μ*^−^
*β*	Small parameter concerning *E*^+^, *E*^−^, *μ*+ and *μ*^−^ shown in Equation (61)
*T*_1_, *T*_2_	Dimensionless quantities of *t*_1_ and *t*_2_
*λ_h_*, *λ_t_*	Dimensionless quantities of *h* and *t*
*K*^+^, *K*^−^	Dimensionless quantities of *D*^+^ and *D*^−^
*Q*_*cr*1_, *Q*_*cr*2_, *Q*_*cr*4_, *Q*_*cr*3_	Dimensionless quantities of *q*_*cr*1_, *q*_*cr*2_, *q*_*cr*3_ and *q*_*cr*4_
